# Prognostic value of resting coronary sinus flow determined by phase-contrast cine cardiovascular magnetic resonance in patients with known or suspected coronary artery disease

**DOI:** 10.1186/s12968-021-00790-9

**Published:** 2021-08-19

**Authors:** Shingo Kato, Kazuki Fukui, Sho Kodama, Mai Azuma, Naoki Nakayama, Tae Iwasawa, Kazuo Kimura, Kouichi Tamura, Daisuke Utsunomiya

**Affiliations:** 1 Department of Diagnostic Radiology, Yokohama City Graduate School of Medicine, Yokohama, Kanagawa Japan; 2 Department of Cardiology, Kanagawa Cardiovascular and Respiratory Center, Yokohama, Kanagawa Japan; 3 Department of Cardiology, Yokohama City University Medical Center, Yokohama, Kanagawa Japan; 4 Department of Medical Science and Cardiorenal Medicine, Yokohama City University, Yokohama, Kanagawa Japan; 5 Department of Radiology, Kanagawa Cardiovascular and Respiratory Center, Yokohama, Kanagawa Japan

**Keywords:** Coronary artery disease, Prognosis, Resting coronary sinus flow, Phase contrast cine

## Abstract

**Background:**

Phase-contrast cine cardiovascular magnetic resonance (CMR) of the coronary sinus has emerged as a non-invasive method to measure coronary sinus blood flow (CSBF) and coronary flow reserve (CFR). We aimed to compare the prognostic value of resting CSBF and CFR for predicting major adverse cardiac events (MACE) in patients with known or suspected coronary artery disease (CAD) who underwent vasodilator stress CMR.

**Methods:**

We studied 693 patients with known CAD and 519 patients with suspected CAD admitted to our hospital between 2009 and 2019. The CFR was calculated as the CSBF during adenosine triphosphate infusion divided by CSBF at rest. MACE was defined as composite of cardiovascular death, acute coronary syndrome, heart failure hospitalization, and sustained ventricular tachyarrhythmia.

**Results:**

During a median follow-up of 4.6 years, 92 patients (8%) experienced MACE. The resting CSBF was significantly higher in patients with MACE than in patients without MACE (114.7 ± 44.9 mL/min vs. 84.7 ± 30.9 mL/min, p < 0.001 for known CAD; 122.2 ± 33.3 mL/min vs. 86.6 ± 36.7 mL/min, p < 0.001 for suspected CAD). The resting CSBF remained a significant predictor for MACE after adjusting clinical and CMR variables (hazard ratio [HR] of resting CSBF higher than the median: 3.18, p = 0.0083 for known CAD; HR: 23.3, p < 0.001 for suspected CAD). The area under the curve for predicting MACE was 0.73 for resting CSBF, 0.72 for CFR (p = 0.78) in patients with known CAD, and 0.82 for resting CSBF, 0.83 (p = 0.58) for CFR in patients with suspected CAD.

**Conclusions:**

The resting CSBF may be a useful non-invasive method for the risk stratification of patients with known or suspected CAD without any radiation exposure, contrast media, or pharmacological vasodilator agents.

## Introduction

Accurate assessment of myocardial ischemia is crucial for improving revascularization in patients with coronary artery disease (CAD). Fractional flow reserve (FFR) has been established to identify physiological ischemia during hyperemia provoked by adenosine triphosphate (ATP); research has revealed that FFR-guided revascularization substantially improves the prognosis of CAD patients [1–3]. Recently, the resting index without the use of pharmacologic agents has been proposed as an alternative method to identify significant CAD. The instantaneous wave-free ratio (iFR) is a representative resting index to perform waveform analysis to detect changes in flow patterns due to stenosis; iFR-guided percutaneous coronary intervention (PCI) is non-inferior to FFR-guided PCI, with respect to clinical outcomes [4, 5].

Blood flow of the coronary sinus can be assessed by phase-contrast (PC) cine cardiovascular magnetic resonance (CMR). The accuracy of coronary sinus blood flow (CSBF) measurements have been validated using myocardial positron emission tomography (PET), [6] which is the gold standard method for calculating myocardial blood flow. Global coronary flow reserve (CFR) can be calculated using the ratio of CSBF during ATP infusion divided by that at rest. CFR is a prognostic marker in patients with known or suspected CAD [7, 8]. A recent study used PET to demonstrate that the resting myocardial flow is a main determinant of CFR; in that study, the resting blood flow was elevated to account for ischemia in CAD patients who underwent revascularization [9]. Based on these findings, we hypothesized that, according to PC cine CMR, the resting CSBF will be elevated in high risk CAD patients and would predict future cardiovascular events.

The purposes of this study were to investigate if the resting CSBF is elevated in CAD patients who experienced adverse events and to compare the prognostic values of the resting CSBF and CFR for patients with known or suspected CAD.

## Methods

### Patients

**Fig. 1:**
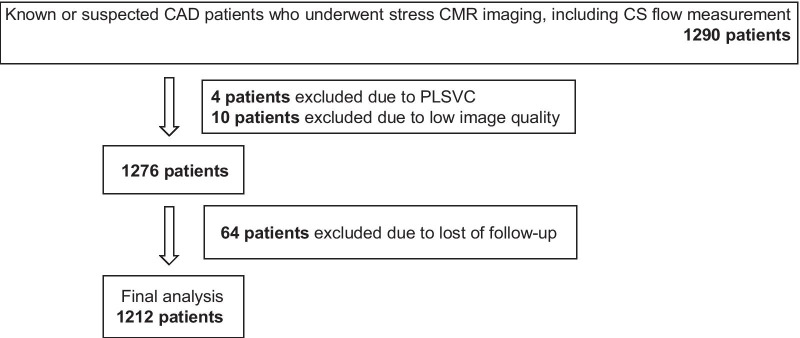
Flow chart of patient selection. *CAD* coronary artery disease, *CMR* cardiovascular magnetic resonance, *PLSVC* persistent left superior vena cava

This retrospective observational study included 1290 patients with known or suspected CAD who underwent vasodilator stress CMR between 2009 and 2019. Known CAD was defined as history of myocardial infarction, previous PCI or coronary artery bypass graft, or angiographically significant coronary artery stenosis (> 70% diameter stenosis in any epicardial coronary artery or > 50% of the left main coronary artery). Suspected CAD was defined as having symptoms suspicious of myocardial ischemia (including chest pain and dyspnea on exertion) or ischemic changes on electrocardiogram (i.e., ST segment depression, abnormal Q waves, inverted T waves). Figure 1 shows the flow chart for patient selection. Four patients were excluded due to having a persistent left superior vena cava. Ten patients were excluded due to low image quality. Follow-up information was obtained from 95% of the population. A total of 1212 patients were included in the final analyses. This study was approved by the institutional review board of our institution which waived informed consent.

### Acquisition of CMR images

Using a 1.5T CMR scanner equipped with 32-channel cardiac coils (Achieva; Philips Healthcare, Best, the Netherlands), data from cine CMR, PC cine of the coronary sinus, stress perfusion CMR, and late gadolinium enhancement (LGE) CMR were acquired. Cine CMR of the left ventricle was acquired using a balanced steady-state free-precession sequence (repetition intervals, 4.1 ms; echo intervals, 1.7 ms; flip angle, 55°; field of view, 350 × 350 mm; acquisition matrix, 128 × 128; slice thickness, 10 mm; number of phases per cardiac cycle, 20). First-pass perfusion CMR images were obtained by a turbo field echo sequence to evaluate the presence and severity of myocardial ischemia (3 short-axis slices/ 1RR interval; repetition duration, shortest; echo duration, shortest; flip angle, 40°; field of view, 360 × 324 mm; acquisition matrix, 192 × 172; reconstruction matrix, 256 × 230; slice thickness, 8 mm). Immediately after beginning the scan for perfusion, 0.05 mmol/kg of gadolinium contrast (gadopentetate dimeglumine, Magnevist; Bayer Healthcare, Berlin, Germany; or gadoterate meglumine, Magnescope; Guerbet, Paris, France; or Gd-BTDO3A, Gadovist; Bayer Healthcare) was injected into the antecubital vein at a flow rate of 4 mL/s. Pharmacological stress was induced by continuous injection of ATP (140 μg/kg/min). The time between the acquisition of stress and resting perfusion CMR was at least 10 min. We evaluated the presence and severity of myocardial infarction or scarring by acquiring LGE images in the same planes as the cine images using inversion recovery-prepared gradient-echo sequences (repetition duration, 4.3 ms; echo duration, 1.3 ms; flip angle, 15°; field of view, 380 × 380 mm; acquisition matrix, 256 × 180; slice thickness, 10 mm). All patients refrained from consuming caffeinated beverages for at least 24 h before the CMR.

### Acquisition of PC cine CMR data from the coronary sinus

**Fig. 2:**
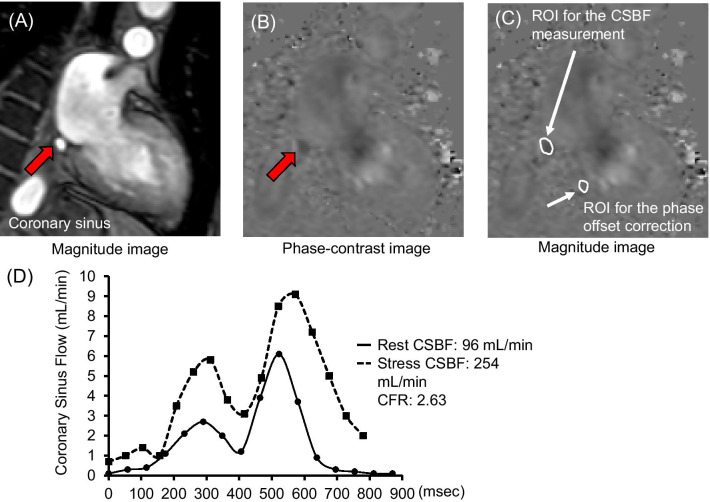
Coronary sinus blood flow measurements. **A**, **B** Phase-contrast cine images of the coronary sinus. **C** Location of ROI for CSBF measurements and phase-offset correction. **D** Representative blood flow in the coronary sinus. *CSBF* coronary sinus blood flow, *ATP* adenosine triphosphate, *ROI* region of interest, *CFR* coronary flow reserve

Figure 2 illustrates PC cine CMR data from the coronary sinus and blood flow curves of the CSBF. The imaging plane for measuring blood flow was set perpendicular to the coronary sinus at 1.5–2.0 cm from its ostium on axial cine CMR images. We acquired PC cine CMR of the coronary sinus while patients were holding their breath (repetition duration, 7.3 ms; echo duration, 4.4 ms; flip angle, 10°; field of view, 380 × 228 mm; acquisition matrix, 160 × 160; reconstruction matrix, 256 × 256; reconstruction resolution, 1.48 × 1.48 mm; number of phases per cardiac cycle, 20; velocity encoding, 50 cm/s; slice thickness, 6 mm) (Fig. 2A, B).

### CMR image analysis

We analyzed cine, perfusion, PC cine, and LGE images using Extended MR WorkSpace workstation (Philips Healthcare). To measure the left ventricular (LV) volume, mass, and ejection fraction (LVEF), we performed manual tracing of the LV epicardial and endocardial borders on short-axis cine images. LV mass was calculated as the sum of the myocardial volume areas multiplied by the assumed specific gravity (1.05 g/mL) of the myocardial tissue [10], To calculate the %LGE, enhanced myocardium was defined as region with > 5SD signal intensity than the remote myocardium [11]. The contours of the coronary sinus were manually traced to quantify the CSBF, and the velocity of the adjacent myocardium was measured to perform phase-offset correction (Fig. 2C) by subtracting the background velocity of the adjacent myocardium from the velocity of the CSBF at each phase. The CSBF was calculated by integrating the product of the cross-sectional area and mean velocity in the coronary sinus.

We calculated the ΔCSBF and CFR using the following equations:

$$\triangle{\text{CSBF }}\left( {{\text{mL}}/{\text{min}}} \right){\text{ }} = {\text{ CSBF during ATP infusion }}\left( {{\text{mL}}/{\text{min}}} \right){\text{ }}{-}{\text{ CSBF at rest }}\left( {{\text{mL}}/{\text{min}}} \right)$$

$${\text{CFR }} = {\text{ CSBF during ATP infusion }}\left( {{\text{mL}}/{\text{min}}} \right){\text{ }}/{\text{ CSBF at rest }}\left( {{\text{mL}}/{\text{min}}} \right).$$

As resting CSBF could be influenced by LV mass and rate pressure product (RPP), we calculated the corrected myocardial blood flow (cMBF) and compare its prognostic value with that of resting CSBF:

$${\text{RPP}} = {\text{systolic blood pressure }} \times {\text{ heart rate}}$$

$${\text{MBF at rest }}\left( {{\text{mL}}/{\text{min}}/{\text{g}}} \right) = {\text{ CSBF at rest }}\left( {{\text{mL}}/{\text{min}}} \right){\text{ }}/{\text{ LV mass }}\left( {\text{g}} \right).$$

$${\text{cMBF at rest }}\left( {{\text{mL}}/{\text{min}}/{\text{g}}} \right) = {\text{MBF at rest }} \times {\text{ RPP }}/{\text{75}}00$$

The average rate pressure product at rest was 7500 from healthy controls with mean age of 50.1 years reported in a previous study [12].

Myocardial ischemia was defined as hypointensity for ≥ 3 frames after peak myocardial enhancement; it was located within the viable myocardium and distributed along the coronary artery territory [13–15]. We used a model including 32 subsegments (endocardial and epicardial sectors for each of the American Heart Association 16-segments) to calculate the percent ischemia. The criterion for categorization as high risk was > 10% ischemia, in accordance with the nuclear sub-study of the COURAGE trial [16]. The manual planimetry method was used to calculate the %LGE.

### Follow-up of adverse events

Prognostic data were obtained via electronic medical records. Major adverse cardiovascular events (MACE) were defined as a composite of cardiovascular death, acute coronary syndrome, hospitalization for heart failure, and sustained ventricular tachyarrhythmia. The time to event was defined as the length of time from the CMR scan to the first event. Patients without MACE were censored at the time of the last-follow-up. Adverse events were investigated by medical personnel who were blinded to all CMR findings.

### Statistical analyses

All statistical analyses were performed using SPSS (version 17.0, Statistical Package for the Social Sciences, International Business Machines, Inc., Armonk, New York, USAL), MedCalc for Windows (version 14.8.1, MedCalc Software, Ostend, Belgium), or R version 3.6.3 (The R Foundation for Statistical Computing, Vienna, Austria). Continuous data were presented as means ± standard deviation, while categorical values were presented as numbers (%). The Shapiro–Wilk test was used to determine normality for each variable. Data were compared using the unpaired t-test for normally distributed values, and the Mann–Whitney U test for non-normally distributed values. The chi-squared test was used to test for significance of categorical variables. Patients were allocated into two groups based on the median resting CSBF value of 80.4 mL/min. Multivariable associations with MACE were determined using Cox proportional hazards regression analysis, and event-free survival stratified by the median resting CSBF were estimated using Kaplan–Meier survival curves. Receiver operating characteristics curve of rest CSBF, CFR, > 10% ischemia on perfusion CMR, cMBF at rest were generated to compare their prognostic value. Significance of differences of area under the curve (AUC) was tested using a Delong’s method. In addition, prognosis of patients with cMBF more than median (0.89 mL/min/g) was compared with that of patients with equal or less than median using a Kaplan Meier analysis. Multivariable Cox regression analysis was performed using two models. In model 1, continuous variables were used for LGE, ischemia, and resting CSBF; categorical variables were used in model 2. The intra- and interobserver reliabilities of the resting CSBF measurements were assessed in 20 patients using Bland–Altman plots. A p-value < 0.05 was considered statistically significant.

## Results

### Patient characteristics

Table 1 summarizes the characteristics of all patients. The mean age was 69 ± 10 years and 76% were male. The primary indications for stress CMR were chest pain (70%), dyspnea (17%), and electrocardiogram abnormalities (8%). The mean LVEF was 60 ± 12%, and LGE was present in 49% of patients. Ischemia was inducible in 43% of patients. Compared with suspected CAD, those with known CAD had a significantly higher prevalence of dyslipidemia and diabetes mellitus; and significantly more were smokers (p < 0.05). Regarding CMR variables, patients with known CAD had a lower LVEF, and a higher prevalence of LGE and ischemia (Table 1).

**Table 1:** Patient characteristics

	All patients N = 1212	Patients with known CAD N = 693	Patients with suspected CAD N = 519	*P-value
Clinical variables				
Age, years	69 ± 10	69 ± 10	68 ± 11	0.37
Male	921 (76%)	554 (80%)	367 (71%)	< 0.005
BMI, kg/m^2^	24 ± 3	24 ± 3	24 ± 4	0.49
Hypertension	823 (68%)	483 (70%)	340 (66%)	0.13
Dyslipidemia	821 (68%)	488 (70%)	333 (64%)	0.021
Diabetes mellitus	366 (30%)	229 (33%)	137 (26%)	0.012
Current smoking	79 (7%)	55 (8%)	24 (5%)	0.021
Blood test				
HbA1c, %	6.0 ± 0.8	6.1 ± 0.9	5.9 ± 0.7	0.005
LDL cholesterol	99 ± 28	94 ± 27	107 ± 30	< 0.001
eGFR	66 ± 16	67 ± 16	66 ± 15	0.42
CMR variables				
LVEDV, mL	131 ± 46	128 ± 42	134 ± 51	0.053
LVESV, mL	57 ± 37	55 ± 33	59 ± 42	0.24
LV mass, g	95 ± 34	92 ± 36	99 ± 31	< 0.001
LVEF, %	60 ± 12	60 ± 12	59 ± 12	0.84
Presence of LGE	591 (49%)	439 (63%)	152 (29%)	< 0.001
LGE, %	7.9 ± 9.0	9.6 ± 9.6	5.9 ± 7.7	< 0.001
Presence of ischemia	516 (43%)	195 (28%)	321 (62%)	0.002
> 10% ischemia	258 (21%)	175 (25%)	83 (16%)	< 0.001

Data are expressed as mean ± SD or number (%)

*P-value represents significance of difference between patients with known CAD and suspected CAD

*BMI* body mass index, *CAD* coronary artery disease, *CFR* coronary flow reserve, *CMR* cardiac magnetic resonance, *eGFR* estimated glomerular filtration rate, *LDL* low-density lipoprotein, *LGE* late gadolinium enhancement, *LV* left ventricular, *LVEDV* left ventricular end-diastolic volume, *LVEF* left ventricular ejection fraction, *LVESV* left ventricular end-systolic volume, *MACE* major adverse cardiac events

### Comparison of the CSBF and CFR between patients with and without MACE

Table 2 compares the CSBF and CFR between patients with and without MACE. The resting CSBF was 86.7 ± 32.9 mL/min in patients with known CAD and 89.8 ± 37.8 mL/min in those with suspected CAD (p = 0.13). The CSBF during stress, ΔCSBF, and CFR did not significantly differ between patients with known CAD and suspected CAD (all comparisons: p > 0.05). Compared with patients without MACE, the resting CSBF was significantly higher in patients with MACE (114.7 ± 44.9 mL/min vs. 84.7 ± 30.9 mL/min, p < 0.001 for known CAD; 122.2 ± 33.3 mL/min vs. 86.6 ± 36.7 mL/min, p < 0.001 for suspected CAD). The CFR was significantly lower in patients with MACE compared to those without MACE (2.27 ± 0.60 vs. 2.89 ± 0.92, p < 0.001 for known CAD; 2.10 ± 0.40 vs. 2.93 ± 0.84, p < 0.001 for suspected CAD) (Table 2). High reproducibility was observed for CSBF measurements (mean difference: 1.1%; limit of agreement: − 3.9 to 6.1% for intra-observer analysis; mean difference: 1.9%; limit of agreement: − 6.3 to 10.1% for inter-observer analysis).

**Table 2:** Comparison of coronary sinus blood flow and CFR

Patients with known CAD	All patients N = 693	Patients without MACE N = 648	Patients with MACE N = 45	*P-value
CSBF at rest (mL/min)	86.7 ± 32.9	84.7 ± 30.9	114.7 ± 44.9	< 0.001
CSBF during ATP infusion (mL/min)	236.4 ± 87.4**	235.8 ± 88.7**	245.3 ± 66.5**	0.47
ΔCSBF (mL/min)	149.7 ± 72.7	151.1 ± 73.8	130.6 ± 50.9	0.068
Coronary flow reserve	2.84 ± 0.91	2.89 ± 0.92	2.27 ± 0.60	< 0.001

Patients with suspected CAD	All patients N = 519	Patients without MACE N = 472	Patients with MACE N = 47	*P-value
CSBF at rest (mL/min)	89.8 ± 37.8	86.6 ± 36.7	122.2 ± 33.3	< 0.001
CSBF during ATP infusion (mL/min)	242.4 ± 87.4**	241.6 ± 90.3**	250.2 ± 48.5**	0.52
ΔCSBF (mL/min)	152.6 ± 70.1	155.1 ± 72.1	127.9 ± 39.2	0.011
Coronary flow reserve	2.85 ± 0.84	2.93 ± 0.84	2.10 ± 0.40	< 0.001

Data are expressed as mean ± SD or number (%)

*P-values represent the difference between patients with and without MACE. **P < 0.05 vs. CSBF at rest

ΔCSBF = CSBF during ATP infusion–CSBF at rest

Coronary flow reserve = CSBF during ATP infusion / CSBF at rest × 100

*ATP* adenosine triphosphate, *CSBF* coronary sinus blood flow, *CFR* coronary flow reserve, *MACE* major adverse cardiac events, *SD* standard deviation

### Prognostic value of resting CSBF for predicting MACE

**Fig. 3:**
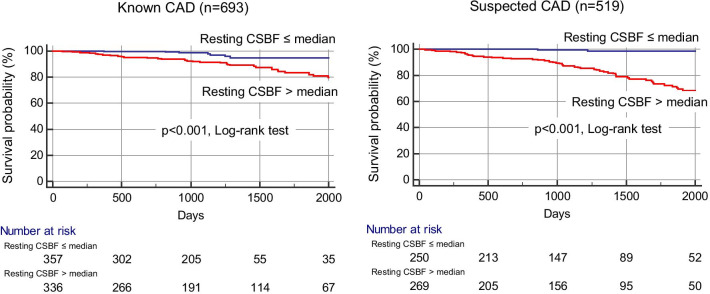
Kaplan–Meier event-free survival curves for patients with major adverse cardiac events stratified by resting CSBF. *CAD* coronary artery disease, *CSBF* coronary sinus blood flow

**Fig. 4:**
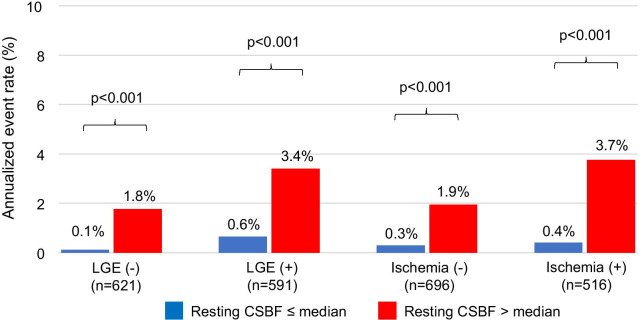
Annualized adverse event rates stratified by rest CSBF according to the presence or absence of LGE or ischemia. *CSBF* coronary sinus blood flow, *LGE* late gadolinium enhancement

Ninety-two (8%) patients experienced MACE over a median follow-up period of 4.6 years (cardiovascular death, n = 29; acute coronary syndrome, n = 38; hospitalization for heart failure, n = 24; sustained ventricular tachyarrhythmia, n = 1). In our cohort, 201 of 258 (78%) patients with > 10% ischemia underwent revascularization by the CMR results. Fifty late revascularization (> 90 days after CMR) were observed including 38 patients with acute coronary syndrome. According to Kaplan–Meier analysis, patients with a resting CSBF higher than the median showed a significantly higher rate of MACE, in both suspected and known CAD (Fig. 3). Figure 4 shows the annualized event rates, which are stratified by the median resting CSBF in the presence or absence of LGE and ischemia. The annualized event rate was significantly higher among patients with rest CSBF higher than the median, regardless of the presence of LGE (0.1% vs. 1.8%, p < 0.001 in the LGE (−) group; 0.6% vs. 3.4%, p < 0.001 in the LGE (+) group). This trend was maintained in the subgroups that were stratified by the presence or absence of ischemia (0.3% vs. 1.9%, p < 0.001 in the ischemia (−) group; 0.4% vs. 3.7%, p < 0.001 in the ischemia (+) group) (Fig. 4).

### Prognostic value of rest CSBF, CFR and stress perfusion CMR

**Fig. 5:**
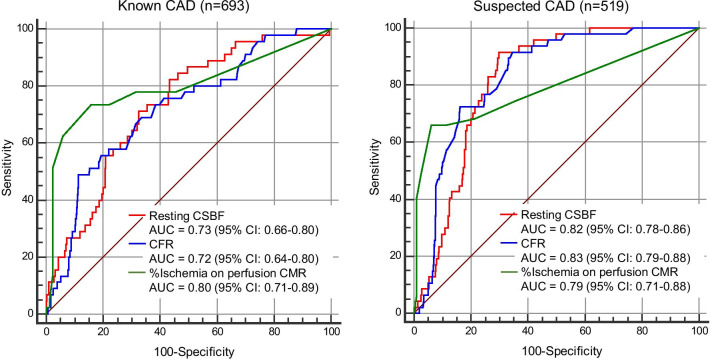
Receiver operating characteristic curves for predicting MACE. *AUC* area under the curve, *CAD* coronary artery disease, *CSBF* coronary sinus blood flow, *CFR* coronary flow reserve, *CI* confidence interval, *MACE* major adverse cardiac events

**Fig. 6:**
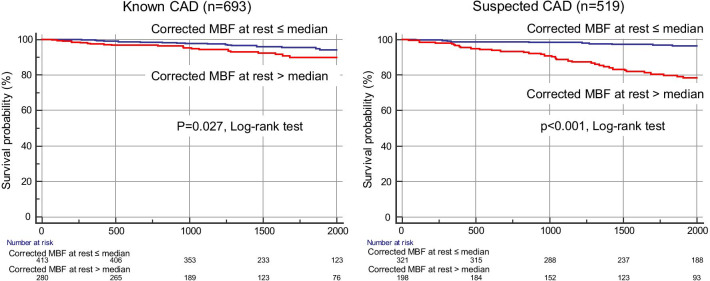
Kaplan–Meier event-free survival curves for patients with major adverse cardiac events stratified by corrected MBF at rest. *CAD* coronary artery disease, *MBF* myocardial blood flow

**Fig. 7:**
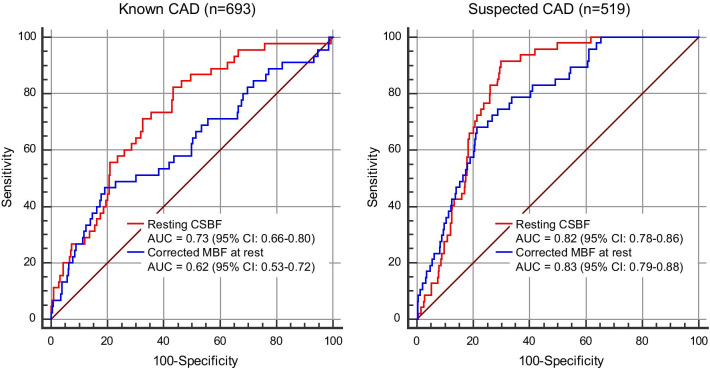
Comparison of receiver operating characteristic curves between resting CSBF and corrected MBF at rest. *AUC* area under the curve, *CAD* coronary artery disease, *CSBF* coronary sinus blood flow, *CI* confidence interval, *MBF* myocardial blood flow

In patients with known CAD, multivariable Cox regression analysis identified age, LVEF, ischemia extent, and resting CSBF as significant prognostic factors for MACE (hazard ratio [HR] of resting CSBF: 1.01 (95% confidence interval [CI]: 1.00–1.02, p < 0.001); Table 3). After converting LGE, ischemia, and resting CSBF to categorical variables, the multivariable model revealed that age, LVEF, ischemia > 10% and a resting CSBF > the median value were independent predictors for MACE (HR of resting CSBF higher than median: 3.18 (95% CI: 1.34–7.51, p = 0.0083); Table 3). In patients with suspected CAD, multivariable Cox regression analysis demonstrated that age, %LGE, ischemia extent, and resting CSBF are significant prognostic factors for MACE (HR of resting CSBF: 1.02 (95% CI: 1.01–1.02, p < 0.001); Table 3). After converting LGE, ischemia, and resting CSBF to categorical variables, the multivariable model revealed that age, LV mass, > 10% ischemia, and resting CSBF higher than the median were independent predictors for MACE (HR of rest CSBF higher than median: 23.3 (95% CI: 5.19–104.9, p < 0.001); Table 3). Figure 5 shows the receiver operating characteristic curves for predicting MACE. AUC of resting CSBF was 0.82 (95%CI: 0.78–0.86) for suspected CAD and 0.73 (0.66–0.80) for known CAD. AUC of resting CSBF and CFR was similar for known and suspected CAD (0.73 vs. 0.72, p = 0.78 for known CAD; 0.82 vs. 0.83, p = 0.58). In patients with known CAD, the AUC of stress perfusion CMR was highest (0.80) among 3 CMR parameters (Fig. 5). Patients with cMBF more than median (0.89 mL/min/g) showed significantly worse clinical outcome compared with those with cMBF equal to or less than median both in suspected and known CAD (Fig. 6). Figure 7 illustrates the comparison of the receiver operating characteristics curves of resting CSBF and cMBF at rest. In suspected CAD, AUC of resting CSBF and cMBF at rest were similar (0.82 vs. 0.83, p = 0.21), however, in known CAD, AUC of cMBF at rest was significantly lower compared to that of resting CBF (0.62 vs. 0.73, p < 0.001).

**Table 3:** Multivariable Cox regression analysis for predicting MACE

	Known CAD N = 693				Suspected CAD N = 519			
	Multivariable model 1*		Multivariable model 2↑		Multivariable model 1*		Multivariable model 2↑	
	Hazard ratio (95% CI)	P-value	Hazard ratio (95% CI)	P-value	Hazard ratio (95% CI)	P-value	Hazard ratio (95% CI)	P-value
Age	1.04 (1.00–1.07)	0.022	1.03 (0.99–1.07)	0.034	1.08 (1.04–1.12)	< 0.001	1.04 (1.00–1.08)	0.023
Male	0.60 (0.25–1.44)	0.25	0.66 (0.26 –1.62)	0.36	1.72 (0.79–3.72)	0.16	2.13 (0.94–4.85)	0.068
Hypertension	0.97 (0.39–2.37)	0.94	0.87 (0.36–2.10)	0.76	1.16 (0.54–2.47)	0.69	1.15 (0.54–2.46)	0.71
Dyslipidemia	0.79 (0.34–1.89)	0.60	0.82 (0.36–1.89)	0.64	0.78 (0.38–1.61)	0.51	0.36 (0.16–0.77)	0.42
Diabetes mellitus	0.92 (0.48–1.80)	0.82	1.49 (0.81–2.73)	0.19	1.50 (0.79–2.84)	0.20	1.17 (0.62–2.23)	0.61
LVEF	0.95 (0.92–0.97)	< 0.001	0.95 (0.92–0.97)	< 0.001	0.98 (0.96–1.00)	0.11	0.99 (0.97–1.00)	0.21
LV mass	0.99 (0.99–1.01)	0.90	0.99 (0.98–1.01)	0.70	0.98 (0.97–1.00)	0.11	0.98 (0.97–1.00)	0.026
Presence of LGE			1.27 (0.48–3.35)	0.62			1.40 (0.68–2.88)	0.35
%LGE	0.98 (0.95–1.02)	0.31			0.96 (0.91–1.00)	0.042		
Ischemia > 10%			4.96 (2.50–9.83)	< 0.001			7.62 (3.69–15.7)	< 0.001
Ischemia extent	1.06 (1.03–1.08)	< 0.001			1.07 (1.04–1.09)	< 0.001		
Rest CSBF > median			3.18 (1.34–7.51)	0.0083			23.3 (5.19–104.9)	< 0.001
Rest CSBF	1.01 (1.00–1.02)	< 0.001			1.02 (1.01–1.02)	< 0.001		

*Model 1 uses LGE, ischemia and CFR as continuous variables. ☨Model 2 uses LGE, ischemia and CFR as categorial variables

*CSBF* coronary sinus blood flow, *CFR* coronary flow reserve, *CI* confidence interval, *CS* coronary sinus, *LGE* late gadolinium enhancement, *LVEF* left ventricular ejection fraction, *MACE* major adverse cardiac events

## Discussion

The main findings of the present study include; (1) resting CSBF is significantly higher in patients with MACE compared with those without MACE both in known and suspected CAD cohorts; (2) the annualized event rate is higher in patients with resting CSBF higher than the median value, irrespective of LGE or perfusion findings; (3) the AUC of resting CSBF is similar to that of CFR for predicting future MACE both in known and suspected CAD. These results suggested that the resting CSBF may be useful as a novel imaging marker for risk stratification for patients with known or suspected CAD without any radiation exposure, contrast media, and pharmacological agents.

In clinical practice, various modalities are used to evaluate myocardial ischemia for patients with CAD; non-invasive methods, such as stress-echocardiography, single-photon emission computed tomography, perfusion CMR, and FFR-computed tomography, and invasive FFR as a gold standard invasive method, have been used to evaluate physiological ischemia [17]. FFR is an attractive method because FFR-guided PCI improves the clinical outcomes of stable CAD patients; however, it is disadvantageous as it requires pharmacological drugs to achieve hyperemia. Recent studies have shown that iFR-guided revascularization is non-inferior to FFR-guided PCI, in terms of outcomes for stable CAD [4, 5]. These data demonstrate the potential clinical importance and utility of resting indices of for the management of stable CAD patients. A recent study used PET to demonstrate the importance of resting coronary blood flow as the main determinant of CFR [9]. That study revealed that resting coronary blood flow may predict successful revascularization. In patients with successful revascularization, the resting flow dropped to 25.0 ± 3.1%; however, peak flow was not significantly changed. These findings indicated that resting coronary blood flow is more important than the peak flow when calculating CFR. Therefore, we conducted this study to evaluate the clinical utility of resting CSBF using PC cine CMR in high risk patients with CAD. An autopsy study demonstrated that the coronary sinus drains approximately 96% of the total myocardium; [18] therefore, the total myocardial blood flow can be estimated by measuring the CSBF. CSBF measurements of the coronary sinus using PC cine CMR are reproducible and are well validated, compared to measurements obtained using PET, [6] phantom models, [19] or flow probes [20]. CFR, the ratio of stress/resting CSBF, is impaired in various cardiovascular diseases, including CAD, [7, 8, 21] cardiomyopathy, [22, 23] and heart failure [24, 25]. In addition, the strong prognostic value of CMR-derived CFR has been demonstrated in patients with CAD [7, 8]. However, there are no known studies that have investigated the prognostic value of resting CSBF in patients with CAD. Our study has shown that the resting CSBF is significantly higher in patients with MACE compared to those without MACE. Therefore, measuring the resting CSBF may be a useful non-invasive imaging method for the risk stratification for known or suspected CAD.

### Clinical implications

Due to its non-invasiveness, measuring the resting CSBF can be useful for young patients or can help prevent side effects of vasodilator agents (such as chest discomfort and hypotension). In addition, measuring the CSBF is a simple and rapid method; therefore, it can be applied in busy clinical laboratories. For this reason, our study findings may impact the screening of CAD using CMR. For instance, the combination of cine CMR (LV volumes and LVEF) and resting CSBF may be useful as simple and non-invasive screening CMR method for CAD. In addition, similar to CFR, resting CSBF was more suitable for predicting MACE in patients with suspected CAD than those with known CAD; this was reflected by a higher AUC for MACE (0.82 vs. 0.73) (Fig. 5). These results indicated that risk stratification using the resting CSBF is useful for the primary prevention of CAD or the early stages of atherosclerosis in patients without overt CAD or angiographically normal coronary arteries. Primary prevention strategies using statin or antiplatelet therapy for suspected CAD patients with a high resting CSBF potentially improve patient outcomes. Further prospective study would be necessary to clarify this point.

Perfusion at rest is mainly determined by the LV demand of oxygen supply. Blood pressure and heart rate are the two main determinants of blood supply demand. Therefore, we calculated corrected cMBF and compared with CSBF in terms of risk stratification. In known CAD, AUC of cMBF was significantly lower compared to resting CSBF (0.62 vs. 0.73, p < 0.001). In suspected CAD, AUC of cMBF was similar to resting CSBF (0.83 vs. 0.82, p = 0.21) (Fig. 7). These results suggested that the resting CSBF may be useful prognostic marker, however, attention should be paid that many confounders may exist such as resting blood pressure, resting heart rate and LV hypertrophy, especially in known CAD patients. From technical aspect, phase offset correction for CS flow measurement was performed in some previous studies. We used the same method of phase offset correction in a previous paper [22]. In another paper, chest wall and skeletal muscle of the back was used for phase offset correction [6]. We’ve measured velocity of skeletal muscle on PC cine images in 20 patients, and mean velocity of skeletal muscle was 0.27 ± 0.20 cm/s (range: 0.02–0.61 cm/s).

### Study limitations

This study has several limitations. First, due to the single-center and retrospective study design, a prospective multicenter study will be required to confirm our observations. Second, this study excluded the patients with metallic devices, including pacemakers and implantable cardioverter defibrillators. Thus, our results are not applicable to these patients. Third, although there was a clear relationship between the resting CSBF and the occurrence of MACE, precise mechanisms explaining the link between resting CSBF and outcomes cannot be determined from this study. Fourth, the coronary sinus is small (8.3 ± 2.5 mm at mid-diastole [18]) and a mobile structure and accurate setting for the slice location are crucial for reliable results; these require high skills and experience. Resting CSBF was very low in some patients (range of rest CSBF: 40.6–316.6 mL/min). The reason for very low CSBF would be multifactorial, presumably small LV size and measurement error of coronary sinus blood flow may be important factors. Accuracy of coronary sinus blood flow measurement would not be sufficient in some cases, due to small size and mobility of CS, and measurement of blood flow from inferior wall may be difficult as middle cardiac vein drains near ostium of the coronary sinus in some cases. Fifth, percentage of inducible ischemia was high as 43% (percentage of ischemia > 10% was 26%). Probably, relatively high rate of history of revascularization of 33% (398/1212 patients) may be one reason for high rate of inducible ischemia. This may bias the results of our study.

## Conclusions

CMR-derived resting CSBF is significantly higher in patients with MACE compared to those without MACE in both known and suspected CAD. The AUC of resting CSBF is high for predicting future MACE both in known and suspected CAD. These results suggested that the resting CSBF may be a useful novel non-invasive resting index that does not involve any radiation exposure, contrast media, and pharmacological stress agents for patients with known or suspected CAD.

## Data Availability

The datasets during and/or analyzed during the current study available from the corresponding author on reasonable request.

## Abbreviations

ATP: Adenosine triphosphate
BMI: Body mass index
CAD: Coronary artery disease
CFR: Coronary flow reserve
cMBF: Corrected myocardial blood flow
CMR: Cardiovascular magnetic resonance
CSBF: Coronary sinus blood flow
FFR: Fractional flow reserve
HR: Hazard ratio
iFR: Instantaneous wave-free ratio
LGE: Late gadolinium enhancement
LV: Left ventricle/left ventricular
LVEDV: Left ventricular end-diastolic volume
LVEF: Left ventricular ejection fraction
LVESV: Left ventricular end-systolic volume
MACE: Major adverse cardiovascular events
MBF: Myocardial blood flow
PC: Phase-contrast
PCI: Percutaneous coronary intervention
PET: Positron emission tomography
PLSVC: Persistant left superior vena cava
RPP: Rate pressure product
